# Giant basal cell carcinoma of the eyelid: a case history

**DOI:** 10.11604/pamj.2016.24.281.10095

**Published:** 2016-07-28

**Authors:** Mohamed Fetohi, Abderrahmane El Mazghi

**Affiliations:** 1 Medical Oncology Department, Military Hospital Moulay Ismaïl, Meknes, Morocco; 2 Radiotherapy Department, CHU Hassan II, Fez, Morocco

**Keywords:** Basal cell, carcinoma, eyelid

## Image in medicine

Basal cell carcinoma is a type of skin cancer and rare, aggressive forms of basal cell carcinoma can invade and destroy nearby muscles, nerves and bone. Very rarely, basal cell carcinoma can spread to other areas of the body. We report the case of a 70-year-old woman who present 3 years ago a small nodule in the right upper eyelid neglected until a very important increase in its volume what motivated a consultation in ophthalmology and in which a biopsy was in favor of an infiltrating basal cell carcinoma. The clinical examination at admission found a patient with poor performance status (3-4) who has a huge ulcerated lesion with open air exposure of the right eye (almost anophthalmia). The lesion extends from the upper right eyelid in the right nasal cavity. The CT scan of the skull shows a skin tumor process allure of 52x44x40 mm, centered on the roof of the right orbit with ipsilateral extension; intracranial and endo sinusienne and an osteolytic lesion of the cranial vault and the temporo-mandibular right joint. The patient received a hemostatic and analgesic palliative radiotherapy of 30 Gy in 10 fractions of 3 Gy. She died 09 months after the end of irradiation in Intensive care unit due to septic shock.

**Figure 1:**
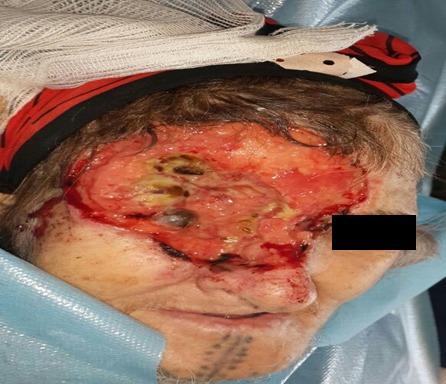
Giant basal cell carcinoma of the eyelid

